# Attentional Bias in Exercise Dependence: An ERP Study of Enhanced Early Attentional Allocation to Exercise Cues

**DOI:** 10.3390/bs16020189

**Published:** 2026-01-28

**Authors:** Yutong Li, Shiyi Ma, Jiangang Li, Xinning Zhou, Jierong Xu, Qianyi Zhang, Hongying Fan

**Affiliations:** 1 School of Psychology, Beijing Sport University, Beijing 100084, China; 2 Key Laboratory of Exercise and Physical Fitness (Beijing Sport University), Ministry of Education, Beijing 100084, China; 3 Laboratory of Sports Stress and Adaptation of General Administration of Sport, Beijing 100084, China

**Keywords:** exercise dependence, N1 potential, attentional bias, dot-probe task, visual attention

## Abstract

Exercise dependence is a maladaptive pattern of excessive exercise characterized by psychological and cognitive symptoms. The existence and nature of attentional bias in individuals with exercise dependence remain unclear. This study combined behavioral measures and event-related potentials (ERPs) to examine the processing of exercise-related cues in this population. The experiment compared exercise-dependent individuals (n = 21) and matched controls (n = 21) using a dot-probe task. Results demonstrated that the exercise dependence group exhibited significantly faster response times in congruent conditions and higher attentional bias scores compared to controls. ERPs data revealed enhanced N1 amplitudes in the exercise dependence group, while no significant group differences were observed in P2 amplitudes. These findings indicate that exercise-related cues automatically capture cognitive resources during the initial stages of attentional processing in dependent individuals. The study provides neurophysiological evidence that may advance the understanding of neurocognitive mechanisms underlying exercise dependence.

## 1. Introduction

While regular exercise provides health benefits, it becomes problematic when escalating into a pattern of psychological dependence. An estimated 3–9% of exercisers develop chronic, compulsive exercise behavior (Marques et al., 2019). Contrary to the benefits of moderate exercise, exercise dependence involves distinct physiological and psychological mechanisms. Exercise dependence is described as a maladaptive pattern of excessive exercise that manifests in psychological and cognitive symptoms such as anxiety and depression (Adams et al., 2003; Hausenblas & Downs, 2002; Schaub et al., 2024; Starcevic & Khazaal, 2017). Also, it could lead to a variety of physical pathologies, including osteoporosis, dysrhythmias, and myocardial fibrosis (Hausenblas et al., 2017).

According to previous studies, exercise dependence can be categorized into primary exercise dependence and secondary exercise dependence (Bamber et al., 2000; Blaydon & Lindner, 2002; Gonçalves Baptista et al., 2019). Primary exercise dependence is characterized by a maladaptive pattern in which excessive exercise itself becomes the central object of dependence, significantly disrupting daily functioning. Motivation is primarily intrinsic, driven by pleasure derived from the activity and avoidance of negative emotional states upon its cessation (Grandi et al., 2011). Clinically, this presentation aligns closely with behavioral addiction frameworks, wherein physical activity is pursued as an end in itself (Hamer & Karageorghis, 2007). In contrast, secondary exercise dependence arises almost exclusively within the context of eating disorders (e.g., anorexia nervosa or bulimia nervosa). Here, compulsive exercise is not an end in itself but serves an instrumental, compensatory role aimed at weight control, caloric purging, or body shape alteration (Landolfi, 2013; Lyvers et al., 2023; Schreiber & Hausenblas, 2015). This form has been described as more compulsive in nature (Cunningham et al., 2016). In such cases, attentional resources may be primarily oriented toward weight-, shape-, or food-related cues, with exercise serving a secondary, instrumental purpose. Accordingly, primary exercise dependence is operationally defined by pathological exercise patterns in the absence of co-occurring eating disorder symptomatology (Gonçalves Baptista et al., 2019; Veale, 1995). The present study focuses specifically on primary exercise dependence, as this approach allows for the investigation of cognitive mechanisms—such as attentional bias—associated with maladaptive exercise while minimizing the confounding influence of eating disorder psychopathology.

Attentional bias refers to the tendency for individuals to selectively allocate more attention to certain stimuli at the expense of others (Robbins & Ehrman, 2004; Waters & Feyerabend, 2000). For instance, emotional stimuli can capture attention, elicit automatic affective responses, and trigger immediate approach or avoidance impulses (Dolcos et al., 2022; Pool et al., 2016). In this framework, stimuli that have been consistently paired with reward can elicit a characteristic pattern of response: an attentional bias toward the cue, a positive implicit attitude, and an approach tendency, which collectively direct information-processing resources in favor of the reward-predicting cues (Sander & Nummenmaa, 2021; van den Berg et al., 2014). A similar bias has been documented in behavioral addictions, such as problematic internet or gaming addiction (Jeromin et al., 2016; Nikolaidou et al., 2019; Refahi et al., 2025). It is therefore plausible that individuals with exercise dependence may also display attentional bias toward exercise-related cues. This automatic cognitive process could maintain or even exacerbate exercise dependence. For example, when an individual attempts to reduce exercise, pervasive environmental cues may trigger intense craving, leading to failed withdrawal attempts. Despite substantial research on attentional bias in substance and behavioral addictions (Anderson, 2016; Brand et al., 2025; Field & Cox, 2008; Heitmann et al., 2018), studies examining this cognitive mechanism in exercise dependence remain limited.

To investigate attentional bias with high temporal precision, researchers often employ event-related potentials (ERPs)—small voltage fluctuations time-locked to specific events. ERPs offer high temporal resolution, allowing fine-grained tracking of the sequential stages of cognitive processing (Fichtenholtz et al., 2007). Early ERP component, N1, which emerges approximately 150–200 ms after stimulus onset, reflects initial automatic capture of attention by cues (Gupta et al., 2019; Omoto et al., 2010). The N1 component is considered a neural correlate of initial attentional orienting (Mei et al., 2024; M.-Y. Wang et al., 2015). The P2 component is sensitive to emotional stimulus content. Enhanced P2 amplitude is thought to reflect both the intensity of emotional evaluation and difficulty in disengaging attention from cues (Bar-Haim et al., 2005). Thus, the P2 may serve as an electrophysiological indicator of attentional engagement and disengagement difficulty (M.-Y. Wang et al., 2015; Wauthia et al., 2023). Behavioral studies on the automatic detection advantage for specific types of stimuli often employ the dot-probe task (Bechor et al., 2019; X. Li et al., 2018; J. Wang & Li, 2024). Attentional bias is inferred when participants respond more rapidly to probes that replace addiction-related stimuli compared to neutral ones. This paradigm, particularly with brief stimulus presentations, allows for the examination of pre-attentive and automatic processing of relevant cues. Combining the dot-probe task with early ERP components—N1 and P2—offers a well-established approach for investigating the temporal dynamics of automatic attentional capture in individuals with exercise dependence.

This study examines attentional bias toward exercise-related cues in exercise dependence. Utilizing event-related potentials (ERPs), we further seek to identify the neurophysiological mechanisms underlying attentional bias. Specifically, we hypothesize that: (1) individuals with exercise dependence will demonstrate a significant attentional bias toward exercise-related cues compared to non-dependent individuals; and (2) ERP data will reveal group differences in early neural responses. Through this approach, we seek to provide electrophysiological evidence clarifying the early-stage cognitive processes involved in exercise dependence.

## 2. Materials and Methods

### 2.1. Participants

Participants were college students with regular exercise habits. A total of 76 subjects were recruited via an online questionnaire, and 42 participants were selected based on inclusion criteria. Based on the methodological reporting guidelines for studies of ERPs (Clayson et al., 2019), a prior power analysis (using G*Power 3.1) determined that at least 28 participants were needed to detect a medium effect (Cohen’s *f* = 0.25, α = 0.05, 1 − β = 0.8), which was satisfied.

All participants were required to meet the following inclusion criteria: (1) native Chinese speaker, (2) right-handed, and (3) no history of any psychiatric disease or disorder. The Exercise Dependence group consisted of 21 participants (11 males, 10 females; age range: 18–28 years, *M_age_* ± *SD* = 22.10 ± 2.61 years). Inclusion criteria included exercising at least 3 times per week for 30 min or more at moderate intensity, a score of ≥53 on the Exercise Dependence Scale (EDS), and ≤20 on the Eating Attitudes Test-26 (Lee, 1993). The Control group also had 21 participants (10 males, 11 females; age range: 18–26 years, *M_age_* ± *SD* = 21.10 ± 2.76 years), meeting the same exercise criteria but scoring <53 on the EDS and ≤20 on the EAT-26. There was no significant age difference between the groups (*p* = 0.234).

### 2.2. Materials

Prior to the experimental session, all participants were requested to complete a set of questionnaires. In addition to the Exercise Dependence Scale (EDS) and the Eating Attitudes Test-26 (EAT-26), basic demographic and behavioral data—including age, gender, and exercise habits—were also collected.

#### 2.2.1. Eating Attitudes Test-26 (EAT-26)

The Eating Attitudes Test-26 (EAT-26) is a widely used self-report measure for assessing symptoms and traits associated with eating disorders (Garner et al., 1982). The Chinese version of the scale comprises three subscales that evaluate eating-related pathology across three dimensions: dieting, bulimia, and oral control (Lee, 1993). The instrument contains 26 items, each of which is categorized into 6 levels: all the time (3 points), always (2 points), often (1 point), occasionally (0 points), rarely (0 points), and never (0 points). Total scores range from 0 to 78, and a cutoff score of 20 or higher indicates potential eating disorder risk, with higher scores reflecting more severe symptomatology. The scale has demonstrated satisfactory reliability and validity in mainland Chinese populations (Kang et al., 2017).

#### 2.2.2. Exercise Dependence Scale (EDS)

The Chinese version of the Exercise Dependence Scale (EDS) was developed based on conceptual framework of exercise dependence (Hausenblas & Downs, 2002), with modifications to align with Chinese cultural contexts (M. Li et al., 2012). The scale comprises 20 items organized across five dimensions: withdrawal symptoms, tolerance, loss of control, excessive exercise behavior, and reduction in other activities. Items are rated on a 5-point Likert scale ranging from 1 (strongly disagree) to 5 (strongly agree). The Chinese version of the EDS demonstrated acceptable internal consistency (Cronbach’s α = 0.793), and a total score of 53 or higher serves as the cutoff for identifying individuals with exercise dependence (M. Li et al., 2012).

#### 2.2.3. Exercise-Related Words

The stimulus materials for the dot-probe task were carefully screened and normed prior to the formal study to ensure effective assessment of attentional bias toward exercise-related cues. The selection procedure aimed to identify exercise-related words that reliably elicit attentional bias as experimental stimuli, while also matching them with neutral words in terms of lexical frequency and structural characteristics.

A total of 43 exercise-related words were initially selected based on existing literature and the *Modern Chinese Dictionary* (7th ed.). To ensure comparability, neutral words were chosen from the *Dictionary of Commonly Used Words in Modern Chinese*, matched to the exercise-related words in lexical frequency with a difference of less than 10%. These words were then evaluated by 17 psychology graduate students (not involved in the main study) across three dimensions—association, familiarity, and generalization—using a 5-point rating scale. Based on the ratings, 30 exercise-related words were finally selected as target stimuli. Independent-samples *t*-tests revealed that the target words were rated significantly higher than neutral words on association and generalization, while no significant difference was found in familiarity. These results support the validity of the selected stimuli for use in the dot-probe task. A detailed comparison between target and neutral words is provided in Table 1.

### 2.3. Dot-Probe Task

Participants were asked to read and sign the Informed Consent Form before the experiment, and then to complete the dot-probe task while wearing an EEG cap. The task comprised three trial conditions: congruent trials, in which the target and neutral stimuli appeared simultaneously with the probe replacing the target; incongruent trials, where the target and neutral stimuli were paired but the probe appeared in the neutral stimulus location; and neutral trials, where both stimuli were neutral.

**Figure 1:**
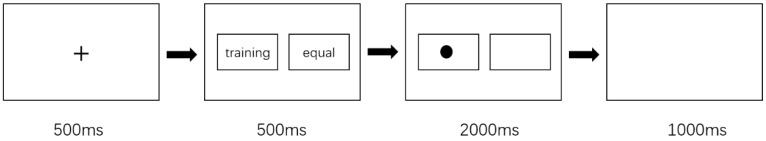
Flowchart of one trial in the dot-probe task.

Each trial followed the sequence illustrated in Figure 1. A central fixation cross was presented for 500 ms, followed immediately by two laterally positioned boxes containing word pairs displayed for 500 ms. After the word pairs disappeared, a probe appeared in the location previously occupied by one of the words. Participants responded to the probe location using the left index finger on the F key for left-side probes and the right index finger on the J key for right-side probes. Word pairs were presented in a randomized and counterbalanced order across left and right positions. Participants were instructed to respond as quickly and accurately as possible. Each probe remained on screen for up to 2000 ms or until a response was detected, followed by a 1000 ms blank screen interval. Based on the established dot-probe research employing ERPs, the experiment included 20 practice trials and 180 experimental trials (Van Dessel & Vogt, 2012; Zhao et al., 2018). Only participants who achieved at least 90% accuracy in the practice phase proceeded to the main experiment. The entire session lasted approximately 15 min.

### 2.4. Apparatus

Stimuli were presented and EEG was recorded on two Lenovo desktops (1920 × 1080 resolution). Experimental programs were controlled by E-Prime 2.0. EEG data were acquired via a Neuroscan 64-channel system. All electrodes were re-referenced to the average of the whole brain. Vertical EOG electrodes were placed above/below the right eye; horizontal EOG at bilateral canthi. Continuous EEG was sampled at 500 Hz (DC-100 Hz bandpass) with electrode impedance maintained below 10 kΩ (Osinsky et al., 2014).

### 2.5. Statistical Analyses

Following established methods (Koster et al., 2006), we derived three attentional components from response times: the Attentional Bias Index (BI), calculated as the difference between incongruent and congruent trials, reflecting overall attentional orientation toward target stimuli; the Attentional Orienting Index (OI), derived from the difference between congruent and neutral trials, indicating engagement speed toward emotional stimuli; and the Attentional Disengaging Index (DI), obtained from the difference between incongruent and neutral trials, representing difficulty in disengaging from target stimuli. These indices collectively captured participants’ attentional processing of exercise-related cues.

Data were preprocessed in EEGLAB. Recordings were band-pass filtered at 1–30 Hz (M.-Y. Wang et al., 2020), bad channels interpolated spherically, and ocular artifacts removed via Independent Component Analysis (ICA). All EEG channels were re-referenced to the average of the two mastoids (M1 and M2). Epochs were extracted (−200 to 800 ms; −200–0 ms baseline corrected). Trials with incorrect responses or EOG artifacts (|amplitude| > 100 μV) were excluded (Sawaki & Luck, 2010). Finally, trials were visually inspected and excluded if EOG artifacts were still observable.

Based on visual inspection of the scalp potential topography, ERPs revealed one prominent negative deflection within the 100–200 ms time window and one prominent positive deflection within the 200–300 ms temporal window. In line with previous ERP studies (Bechor et al., 2019; Zhang et al., 2008), and given our focus on regional rather than individual electrode effects, we focused primarily on the N1 and P2 components over the parietal-occipital region (PO3, POz, and PO4). In accordance with established ERP methodological guidance (Clayson et al., 2013), component amplitudes were quantified using an adaptive mean approach. For each component, we selected a 40 ms interval centered on the peak amplitude (20 ms before to 20 ms after the peak) and computed the average waveform amplitude within this period. Specifically, the N1 component was identified in the 130–170 ms window, and the P2 component in the 230–270 ms window (Job et al., 2019; Olofsson et al., 2011; Turconi et al., 2004).

Independent samples *t*-tests assessed the EAT-26 and EDS score differences between Exercise Dependence and Control groups. Behavioral (reaction times, correctness, attentional bias index) and electrophysiological (N1 and P2) responses were analyzed using ANOVA (α = 0.05). Greenhouse-Geisser corrections addressed sphericity violations; Bonferroni tests handled multiple comparisons.

## 3. Results

### 3.1. Questionnaire Results

#### 3.1.1. Eating Attitudes Test-26 Results

An independent-samples *t*-test on EAT-26 scores revealed no significant difference between the Exercise Dependence group (*M* ± *SD* = 6.43 ± 3.04) and the Control group (*M* ± *SD* = 6.14 ± 4.66, *p* = 0.815). Importantly, all participants obtained EAT-26 total scores below the clinical cutoff of 20, which confirms that the observed exercise dependence patterns occurred in the absence of co-occurring eating disorder symptoms, consistent with the criteria for primary exercise dependence.

#### 3.1.2. Exercise Dependence Scale Results

The *t*-test result of EDS score of Exercise Dependence (*M* ± *SD* = 62.9 ± 5.74) and Control group was significant (*M* ± *SD* = 41.81 ± 10.53; *t* = 8.063, *df* = 40, *p* < 0.001).

### 3.2. The Dot-Probe Task Results

**Figure 2:**
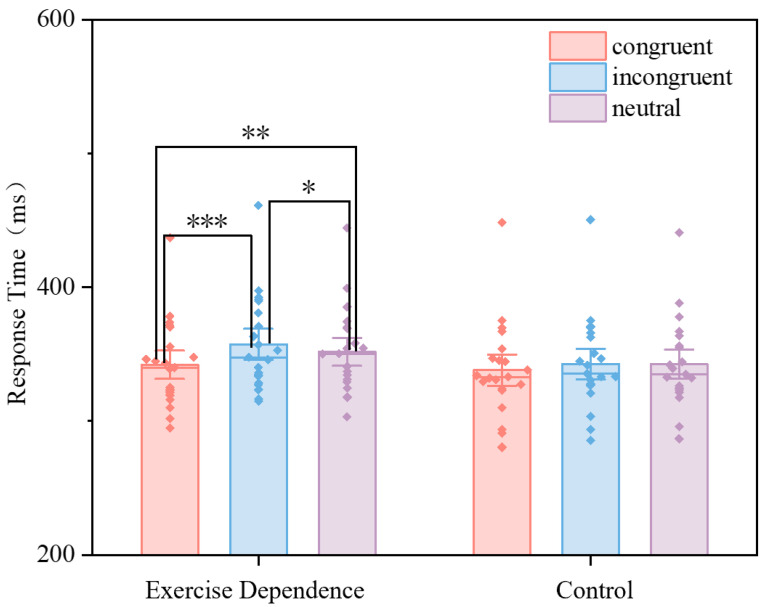
The repeated-measures ANOVA results for the Exercise Dependence and Control groups on the dot-probe task. Bars represent mean ± standard error of the mean response time, * *p* < 0.05, ** *p* < 0.01, *** *p* < 0.001.

A repeated-measures ANOVA was conducted on response times with group (Exercise Dependence vs. Control) as the between-subjects factor and probe location (congruent, incongruent, neutral) as the within-subjects factor, as shown in Figure 2. Descriptive statistics for response times across conditions are summarized in Table 2. Significant main effects of probe location and interaction effect of group and probe location emerged. No significant group main effects were found, as shown in Table 3. Simple effects analysis further showed that within the Exercise Dependence group, response times in the congruent condition (*M* ± *SD* = 341.15 ± 32.19 ms) were significantly shorter than those in both the incongruent condition (*M* ± *SD* = 357.46 ± 35.33 ms, *p* < 0.001) and the neutral condition (*M* ± *SD* = 351.8 ± 31.82 ms, *p* = 0.001). Additionally, responses in the incongruent condition were significantly slower than those in the neutral condition (*p* = 0.037). In contrast, the Control group showed no statistically significant differences in reaction times across the three probe conditions.

A repeated-measures ANOVA was done on subjects’ correctness, where group was the between-subjects variable and probe location was the within-subjects variable. No significant main effects of group and probe location, and interaction effect of group and probe location were found, as shown in Table 3.

### 3.3. Attentional Bias Index Results

**Figure 3:**
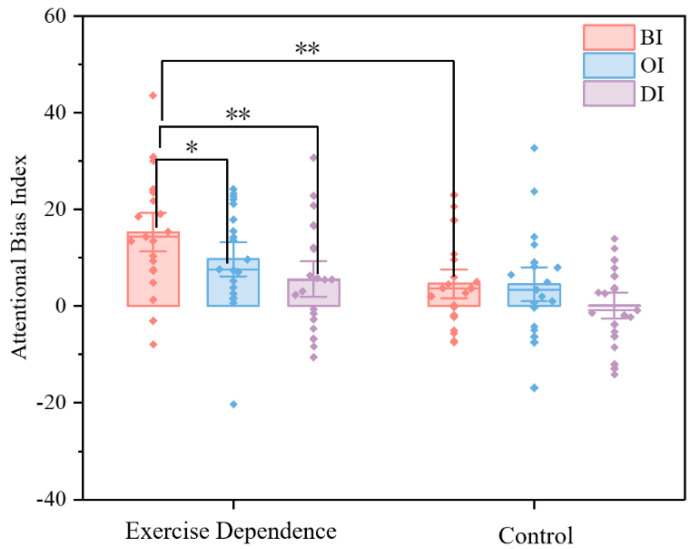
The repeated-measures ANOVA results for the Exercise Dependence and Control groups on the attentional bias index. Bars represent mean ± standard error of the mean score, * *p* < 0.05, ** *p* < 0.01. (BI = bias index, OI = orienting index, DI = disengaging index).

A 2 (group: Exercise Dependence vs. Control) × 3 (attention bias index: BI, OI, DI) repeated measures ANOVA was done on the subjects’ scores on the attention bias index, where group was the between-subjects variable and attention bias index was the within-subjects variable, as shown in Figure 3. The indexes for the Exercise Dependence and Control groups in the different conditions are summarized in Table 2. The results revealed a significant main effect of group, with the Exercise Dependence group (*M* ± *SD* = 10.21 ± 1.55) scoring significantly higher than the Control group (*M* ± *SD* = 3.11 ± 1.55, *p* = 0.002). A significant main effect of attention bias index was also observed. Within the Exercise Dependence group, BI (*M* ± *SD* = 9.99 ± 1.64) was significantly higher than the DI (*M* ± *SD* = 2.87 ± 1.52, *p* < 0.001), while no other significant differences were found between BI and OI, or between OI and OI. The interaction effect of group and attention bias index was not significant (see Table 3).

### 3.4. EEG Analysis Results

**Figure 4:**
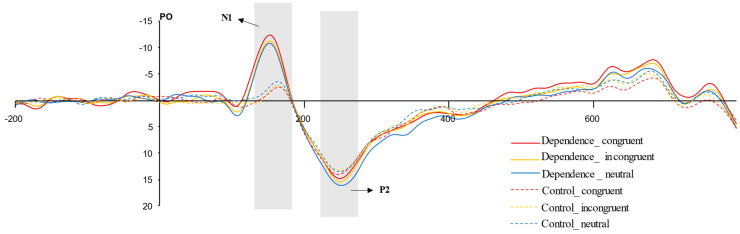
ERPs results of N1 and P2 for both groups under each condition. The PO Waveforms are averaged across PO3, Poz and PO4.

**Figure 5:**
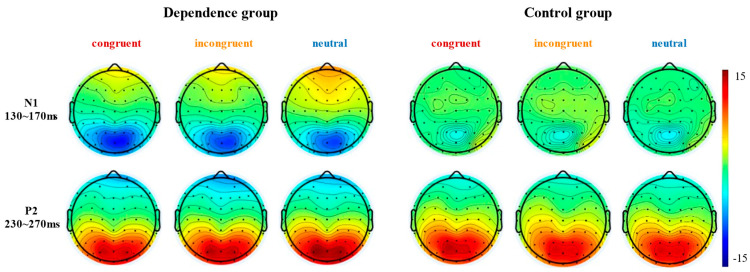
Topographical maps of N1 and P2 components for both groups under each condition.

As depicted in Figure 4, the N1 and P2 components were successfully evoked by stimuli across all three valence conditions. The topographical distribution of the EEG activity for both groups was presented in Figure 5.

A 2 (group: Exercise Dependence vs. Control) × 3 (probe location: congruent, incongruent, neutral) repeated-measures ANOVA was done on the mean amplitude of the N1 and P2 components, respectively, where group was the between-subjects variable and probe location was the within-subjects variable.

The ANOVA on N1 mean amplitude showed significant main effects of group and interaction effect of group and probe location, as shown in Table 3. N1 amplitude was significantly higher in the Exercise Dependence group (*M* ± *SD* = −9.18 ± 1.67 μV) than in the Control group (*M* ± *SD* = −1.50 ± 1.67 μV, *p* = 0.002). A further simple effects analysis revealed that N1 amplitude in congruent condition (*M* ± *SD* = −10.11 ± 7.63 μV) was significantly more negative than in neutral condition (*M* ± *SD* = −8.49 ± 6.75 μV, *p* = 0.02) within the Exercise Dependence group, but not significantly different under other conditions. No significant probe location main effects were found, as shown in Table 3. However, P2 amplitude showed no significant main effects of group, probe location, or interaction effect of group and probe location, as shown in Table 3.

## 4. Discussion

This study aimed to explore the mechanisms of attentional bias within the realms of exercise dependence. Utilizing objective performance metrics rather than subjective self-report method, we directly assessed attentional processing patterns. To our knowledge, this study provides initial evidence through the use of event-related potentials (ERPs) to examine attention bias toward exercise-related stimuli among exercise dependence.

Consistent with Hypothesis 1, results indicated that individuals with exercise dependence exhibited a significant attentional bias toward exercise-related cues, as evidenced by shorter reaction times in the congruent condition of the dot-probe task. In contrast, the control group did not demonstrate a comparable level of attentional bias, further substantiating the presence of a distinct cognitive pattern in exercise dependence. These findings align with prior research on exercise dependence and attentional bias (Brand et al., 2025; Field & Cox, 2008; Thomsen et al., 2014). According to the Incentive-Sensitization Theory, cues associated with addictive behaviors acquire distinctive incentive-motivational properties, thereby guiding behavior toward reward (Berridge & Robinson, 2016; Field & Cox, 2008). The capacity of incentive-predictive cues to direct behavior toward themselves has been documented in Pavlovian conditioning studies (Boakes, 2021). The mechanism underlying cue reactivity may reflect classical conditioning, whereby a neutral stimulus (conditioned stimulus) repeatedly paired with a rewarding stimulus (unconditioned stimulus) eventually elicits a conditioned response, such as physiological arousal. This process contributes to a behavioral pattern characteristic of addiction—driven by a conditioned urge to re-experience sensations triggered by such cues (Robinson & Berridge, 2001). Although initially developed to explain substance addiction, the incentive-sensitization model has since been effectively extended to behavioral addictions such as gambling and internet use (Thomsen et al., 2014; Wegmann & Brand, 2020). In the context of exercise dependence, this framework offers a plausible account for why individuals persist in excessive exercise patterns despite adverse consequences. Exercise-related cues automatically capture attention and elicit approach tendencies. This pattern mirrors the processes observed in both substance-related (Cofresí et al., 2022; Della Libera et al., 2019) and other behavioral addictions (Sklenarik et al., 2019; Thomsen et al., 2014), supporting the relevance of incentive-sensitization mechanisms across various forms of dependence. Furthermore, attentional bias toward addiction-related stimuli can elevate the probability that dependent individuals will engage in the addictive behavior both currently and in the future (Cox et al., 2002). For example, individuals with alcohol dependence may be more inclined to approach and remain in alcohol-related environments, such as bars, because these settings themselves become attractive—not merely as signals for alcohol access and consumption. Similarly, individuals with exercise dependence may find themselves automatically noticing exercise-related cues even during periods of rest, thereby being prompted to exercise more frequently.

Consistent with Hypothesis 2, the ERP data revealed distinctive neural processing patterns in the exercise dependence group. Specifically, the N1 component exhibited significantly enhanced negativity across experimental conditions compared to the control group. These amplified N1 amplitudes in response to exercise-related cues indicate heightened early attentional engagement, reflecting the increased salience of such stimuli. Furthermore, the exercise dependence group demonstrated significantly greater N1 amplitudes in the congruent condition compared to both incongruent and neutral conditions. This enhanced neural response was condition-specific, occurring only when attentional allocation to the cue location directly facilitated behavioral performance. Within the framework of limited cognitive resources, when exercise-related and neutral stimuli compete for attentional priority, individuals automatically allocate greater processing resources to the motivationally salient exercise cues (Gonçalves Baptista et al., 2019). This automatic orienting toward personally relevant stimuli illustrates how attentional bias becomes functionally embedded in the cognitive architecture of exercise dependence, potentially reinforcing maladaptive behavioral patterns through preferential processing of addiction-related cues (Asmaro et al., 2014; Yan et al., 2009). The discrimination of addiction-related cues occurs during early stages of stimulus processing (Nijs et al., 2010; J. Wang et al., 2021; Yang et al., 2015). This bottom-up mechanism reflects an automatic, stimuli-driven attentional capture, where such cues preferentially engage cognitive resources from the initial processing stage.

Contrary to our hypothesis, the P2 component, though modulated, did not yield significant group differences. This suggests that attentional bias in individuals with exercise dependence may be primarily manifested during the initial orienting stage, rather than reflecting difficulties in attentional disengagement (M.-Y. Wang et al., 2015). The P2 component is typically associated with the preliminary categorization and structural encoding of stimulus features (Luck & Hillyard, 1994). The lack of significant amplitude differences between groups at this stage may indicate that the basic semantic information of exercise-related cues is processed at a similar level by both the exercise dependence and control groups during early stimulus evaluation. This pattern further supports the notion that exercise dependence may possess distinctive characteristics. Unlike addictive behaviors involving immediate rewards (e.g., gambling), the rewards associated with exercise are typically delayed. For instance, the intrinsic pleasure derived from exercise—often mediated by neurochemicals such as endocannabinoids—usually emerges only after approximately 30 min of moderate-intensity activity (Raichlen et al., 2013). Such delayed rewards are frequently socially reinforced and culturally valued (e.g., as markers of “diligence” or “long-term planning”), which may mask their addictive potential. Consequently, exercise dependence may constitute a unique form of behavioral addiction, potentially underpinned by physiological and cognitive mechanisms that differ from those involved in other addictive behaviors. Future studies should aim to clarify the distinct neural patterns associated with immediate versus delayed rewards across various types of behavioral addictions. A notable limitation of this study is that all participants were recruited from a sports university. Even individuals in the control group are likely to maintain an above-average baseline level of physical activity and familiarity with exercise-related contexts. This common background may have led to a degree of attentional bias toward exercise cues across both groups, potentially attenuating observable group differences. Therefore, the present results should be interpreted with appropriate caution, and future research should seek to replicate these findings in more generalized populations.

By localizing attentional bias to an early processing stage, this study identifies a potential neural marker and target for the intervention of exercise dependence. However, research in this field continues to face methodological challenges. The reliability of scale-based assessments remains limited, partly due to individual differences in how respondents interpret questions (Szabo et al., 2015)—an issue further compounded by the fact that exercise dependence has not yet been included in major diagnostic frameworks such as the DSM-5. To improve assessment accuracy, future studies should incorporate qualitative methods, such as in-depth clinical interviews. Additionally, prospective research is needed to clarify the causal relationship between neurocognitive changes and the development of exercise dependence. Concurrently, we recognize several limitations inherent to this study. Firstly, our sample was confined to non-clinical populations, potentially limiting the universal applicability of our results. Future research would benefit from exploring attentional bias within clinical samples and including more diverse demographic groups to examine how factors such as age, gender, and exercise background moderate attentional patterns. Second, this study did not disaggregate participants by severity of exercise dependence, which could have revealed more granular relationships between symptom intensity and cognitive markers. Subsequent studies could adopt stratified sampling based on EDS scores to clarify how attentional bias manifests across different levels of exercise dependence. Third, the use of verbal stimuli (word-based dot-probe) presents specific constraints. While effective for assessing attention to conceptually relevant cues, text-based cues may lack the ecological validity of more dynamic stimuli, such as pictures or videos. Future studies could employ ecologically valid visual stimuli or adopt multimodal approaches to compare and cross-validate the attentional bias effects captured by different stimulus formats.

## 5. Conclusions

This study demonstrates that individuals with exercise dependence show attentional bias toward exercise-related cues, as evidenced by both behavioral measures and ERP recordings. The amplified N1 component reflects heightened early attentional engagement with exercise stimuli. These findings suggest that exercise-related cues automatically capture cognitive resources during initial processing stages in dependent individuals.

## Figures and Tables

**Table 1:** Descriptive statistics (*M* ± *SD*) and *t*-test results for each dimension of target and neutral stimulus words.

	Target Words (n = 30)	Neutral Words (n = 30)	*t*	*df*	*p*
Associativity	4.27 ± 0.38	1.59 ± 0.36	27.898	58	<0.001
Familiarity	4.50 ± 0.25	4.44 ± 0.20	1.149	58	0.255
Generalization	4.15 ± 0.28	1.77 ± 0.38	27.852	58	<0.001

**Table 2:** Descriptive statistics (*M* ± *SD*) of reaction times and attentional bias index for both groups. (ED = Exercise Dependence, BI = bias index, OI = orienting index, and DI = disengaging index).

	Reaction Times (ms)			Attentional Bias Index		
	Congruent	Incongruent	Neutral	BI	OI	DI
ED group	342.15 ± 32.19	357.46 ± 35.33	351.84 ± 31.82	15.31 ± 12.06	9.69 ± 10.69	5.62 ± 11.23
Control group	338.09 ± 35.27	342.75 ± 34.65	342.63 ± 33.39	4.66 ± 9.04	4.54 ± 10.84	0.12 ± 8.18

**Table 3:** The repeated-measures ANOVA of the behavioral and ERPs results for both groups.

Dimensions		*SS*	*df*	*MS*	*F*	*p*	*ηp* ^2^
Reaction Times	Probe location	2220.789	2	1110.394	20.430	0.000	0.338
	Group	2738.123	1	2738.123	0.825	0.369	0.020
	Probe location × Group	595.995	2	297.998	5.483	0.006	0.121
correctness	Probe location	0.000	2	0.000	0.600	0.551	0.015
	Group	0.001	1	0.001	3.221	0.080	0.075
	Probe location × Group	0.001	2	0.000	2.485	0.090	0.058
Attentional bias index	Attention bias index	1075.928	1.236	870.174	6.160	0.012	0.133
	Group	1588.980	1	1588.980	10.493	0.002	0.208
	Attention bias index × Group	199.222	1.236	161.124	1.141	0.304	0.028
N1	Probe location	6.881	1.655	4.158	0.822	0.424	0.020
	Group	1856.610	1	1856.610	10.552	0.002	0.209
	Probe location × Group	34.908	1.655	21.093	4.169	0.026	0.094
P2	Probe location	8.735	2	4.367	1.193	0.309	0.029
	Group	69.601	1	69.601	0.413	0.524	0.010
	Probe location × Group	19.234	2	9.617	2.627	0.079	0.062

## Data Availability

The datasets generated and analyzed during the current study are available from the corresponding author on reasonable request.

## Abbreviations

The following abbreviations are used in this manuscript:

ERPs	Event-Related Potentials
EDS	Exercise Dependence Scale
EAT-26	Eating Attitudes Test-26
BI	Attentional Bias Index
OI	Attentional Orienting Index
DI	Attentional Disengaging Index
